# R-Spondin 3 Regulates Mammalian Dental and Craniofacial Development

**DOI:** 10.3390/jdb9030031

**Published:** 2021-08-12

**Authors:** Krishnakali Dasgupta, Jeffry M. Cesario, Sara Ha, Kesava Asam, Lindsay J. Deacon, Ana H. Song, Julie Kim, John Cobb, Jeong Kyo Yoon, Juhee Jeong

**Affiliations:** 1 Department of Molecular Pathobiology, New York University College of Dentistry, New York, NY 10010, USA; krishnakali.dasgupta@gmail.com (K.D.); jeffry.cesario@gmail.com (J.M.C.); smh9813@nyu.edu (S.H.); kra277@nyu.edu (K.A.); ljd306@nyu.edu (L.J.D.); heejaesong95@gmail.com (A.H.S.); yk2182@nyu.edu (J.K.); 2 Department of Biological Sciences, University of Calgary, Calgary, AB T2N 1N4, Canada; jacobb@ucalgary.ca; 3 Soonchunhyang Institute of Medi-Bio Science, Soonchunhyang University, Cheonan 31151, Korea; yoonje1@gmail.com; 4 Department of Integrated Biomedical Science, Soonchunhyang University, Cheonan 31151, Korea

**Keywords:** tooth, embryos, craniofacial development, WNT signaling pathway, *Rspo2*, *Rspo3*, mice

## Abstract

Development of the teeth requires complex signaling interactions between the mesenchyme and the epithelium mediated by multiple pathways. For example, canonical WNT signaling is essential to many aspects of odontogenesis, and inhibiting this pathway blocks tooth development at an early stage. R-spondins (RSPOs) are secreted proteins, and they mostly augment WNT signaling. Although RSPOs have been shown to play important roles in the development of many organs, their role in tooth development is unclear. A previous study reported that mutating *Rspo2* in mice led to supernumerary lower molars, while teeth forming at the normal positions showed no significant anomalies. Because multiple *Rspo* genes are expressed in the orofacial region, it is possible that the relatively mild phenotype of *Rspo2* mutants is due to functional compensation by other RSPO proteins. We found that inactivating *Rspo3* in the craniofacial mesenchyme caused the loss of lower incisors, which did not progress beyond the bud stage. A simultaneous deletion of *Rspo2* and *Rspo3* caused severe disruption of craniofacial development from early stages, which was accompanied with impaired development of all teeth. Together, these results indicate that *Rspo3* is an important regulator of mammalian dental and craniofacial development.

## 1. Introduction

Tooth development in mammals is guided by reciprocal and dynamic interactions between the oral ectoderm and the underlying neural crest-derived mesenchyme. These interactions are mediated by many secreted signaling molecules and transcription factors, which are expressed in specific patterns. Signaling molecules involved in the process include fibroblast growth factors (FGF), bone morphogenetic proteins (BMP), wingless-type MMTV integration site family (WNT), and sonic hedgehog (SHH). The transcription factors involved include members of msh homeobox (MSX), distal-less homeobox (DLX), paired box (PAX), and LIM homeobox proteins (LHX) [1,2,3].

The first morphological sign of tooth development appears as localized thickening of the oral ectoderm (dental lamina) at the 11th day of mouse gestation (E11). The epithelium further thickens and invaginates into the mesenchyme to form a dental placode (E12) and then a bud (E13), with the mesenchyme cells condensing around the bud. Subsequently, a signaling center called an enamel knot is established in the epithelium, and it directs further morphogenesis into the cap stage (E14.5) and the bell stage (E16.5). Differentiation of ameloblasts and odontoblasts from the epithelium and the mesenchyme, respectively, occurs during the late bell stage (E18.5) [1,2,3].

WNT signaling can be divided into the canonical pathway and non-canonical pathways. A large number of studies have demonstrated that canonical WNT signaling is a crucial regulator of tooth development both at early stages (up to the cap stage) and late stages, while the role of non-canonical WNT signaling appears to be restricted to the late stages [4,5,6,7]. In the canonical WNT pathway, WNT ligands bind to the heterodimers of Frizzled (Fz) family and low-density lipoprotein receptor-related protein (LRP) family receptors. Intracellular signal transduction results in the stabilization and nuclear localization of β-catenin, which forms a complex with T cell transcription factors (TCFs) or lymphoid enhancer binding factor 1 (LEF1) to regulate the transcription of target genes [8]. Because canonical WNT ligands are expressed in the epithelium but not in the mesenchyme during tooth development [9], this pathway is thought to mediate intra-epithelial signaling and epithelium-to-mesenchyme signaling [1,10]. Inhibition of the canonical WNT signaling resulted in the arrest of incisor development at the placode stage and molar development at the bud stage [6]. Deletion of *Lef1* also inhibited tooth development beyond the bud stage [11,12].

WNT signaling is regulated by multiple extracellular antagonists and co-activators [13]. R-spondins (RSPOs), which are cysteine-rich secreted glycoproteins, can either inhibit or potentiate WNT signaling depending on the context [14,15]. There are four members of *Rspo* genes in mammals, *Rspo1* through *Rspo4*. RSPOs mainly bind to leucine-rich repeat containing G-protein coupled receptors (LGR), although RSPO2 also has LGR-independent functions [16,17]. *Rspo* genes regulate the development of multiple organs in embryos and stem cells in adults [15,18]. Among them, *Rspo2* was shown to regulate craniofacial development. Mouse mutants for *Rspo2* exhibited partially penetrant cleft lip and cleft palate, hypoplasia of the dentary bone, and diastema teeth on the lower jaw [19,20,21]. However, one study noted that *Rspo2* mutants showed no significant anomalies in the molars and incisors forming at their normal positions [20], and there has not been any other report on a dental phenotype of mouse *Rspo* mutants. A recent paper showed that *rspo2* and *rspo3* regulated tooth numbers in fish [22], but they also highlighted significant differences between fish and mice in *Rspo* expression patterns. Therefore, it remains unknown whether *Rspo* genes are required for tooth development in mammals.

In this study, we examined mouse *Rspo3* mutants and *Rspo2*; *Rspo3* double mutants to demonstrate that *Rspo3* is an important regulator of dental and craniofacial development.

## 2. Materials and Methods

### 2.1. Animals

All the experiments involving mice were performed following a protocol approved by the New York University Institutional Animal Care and Use Committee. *Wnt1-Cre* and *Rspo3* floxed alleles (*Rspo3^fl^*) have been described [23,24]. An *Rspo3* null allele (*Rspo3^−^*) was obtained by crossing *Rspo3^fl^* mice with *CMV-Cre* (Jackson Laboratory, stock 006054) for germline recombination. An *Rspo2*-null allele (*Rspo2*^−^) was generated by the targeted deletion of a half of exon1 and the entire exon2, where exon2 contained the translation start codon. The mice were of a mixed background of C57Bl6, 129, and CD-1.

Wnt1-Cre;Rspo2^+/−^;Rspo3^+/−^ males were mated with Rspo2^+/−^;Rspo3^fl/fl^ females to generate Wnt1-Cre;Rspo3^fl/−^;Rspo2^+/+^ mutants (Rspo3^NCKO^), Rspo2^−/−^;Rspo3^fl/+^ mutants (Rspo2^KO^), and Wnt1-Cre;Rspo2^−/−^;Rspo3^fl/−^ mutants (Rspo2^KO^;Rspo3^NCKO^). Some Rspo3^NCKO^ mutants were also obtained from crosses between Wnt1-Cre;Rspo3^+/−^ males and Rspo3^fl/fl^ females. For the analyses of Rspo3^NCKO^ and Rspo2^KO^ mutants, littermates with at least three functional copies of Rspo2 and Rspo3 (including Rspo3^fl^) were used as controls. For the analysis of Rspo2^KO^;Rspo3^NCKO^ mutants, double heterozygote littermates were also included in controls. The sex of the samples was not determined.

### 2.2. Micro-Computed Tomography (MicroCT)

MicroCT of the mouse head was performed with SkyScan 1172 at 8.55 μm resolution, 55 kV, and 181 μA. 3D reconstruction was performed using the NRecon program from SkyScan.

### 2.3. Preparation of Tissue Sections, Histological Staining, and RNA In Situ Hybridization

The head of perinatal animals was fixed in 4% paraformaldehyde, embedded in paraffin, sectioned at 8 μm, and stained with hematoxylin and eosin. Younger samples were fixed in 4% paraformaldehyde, embedded at optimal cutting temperature (OCT), sectioned at 10–16 μm, and stained with hematoxylin and eosin.

OCT-embedded frozen sections from above were also used for RNA in situ hybridization, performed as described before [25]. All RNA in situ hybridization was performed, and serial sections spanning the entire anterior–posterior axis (for coronal sections) or medial–lateral axis (for sagittal sections) of the oral cavity were examined. The sections were separated by approximately 60–80 μm (E11.5–E13.5), 100 μm (E14.5), or 180 μm (E18.5). We examined each gene in at least three mutant/control pairs per stage, with each pair processed simultaneously, and stated a change in expression only if the results were consistent in all pairs, unless indicated otherwise.

### 2.4. Volume Measurement of the Tooth Bud

Frozen sections (10 μm) of the head were prepared as described above. An entire set of sections through an incisor bud was processed by immunofluorescence (described in the Supplementary Materials) and counterstained with DAPI for nuclei. The tooth bud was manually traced in the fluorescence pictures while blind to the genotype. Tooth bud images were combined into a stack in FIJI [26] and aligned by the StackReg plugin [27]. Volume rendering, surface smoothing, and volume measurement were performed using 3D Slicer [28]. The *p*-value was calculated by two-tailed Student’s *t*-test in Excel. Power was calculated using G*Power software.

## 3. Results and Discussion

### 3.1. Rspo3 Is Essential to the Development of the Lower Incisor

A previous study showed the expression of *Rspo* genes during early development of the molars, but not the incisors [20]. Therefore, we examined the expression of all four *Rspo* genes at E11.5–E14.5 in the incisors (Figure S1). We confirmed that the expression of *Rspo2* and *Rspo3* was specific to the mesenchyme in the incisors, as shown in the molars [20]. In particular, *Rspo3* was expressed in the posterior mesenchyme of the incisors at all stages examined.

**Figure 1:**
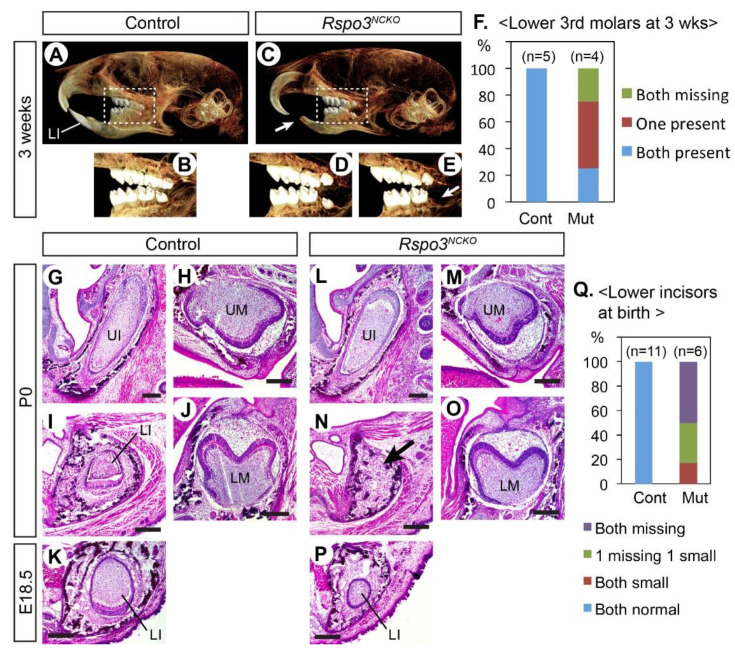
Dental phenotype of *Rspo3^NCKO^* mutants. (**A**–**E**) Micro-CT images of the head. A total of 9 animals were examined (5 controls and 4 mutants). Arrows in (**C**,**E**) point to the missing teeth. (**F**) Summary of the lower third molar phenotype. (**G**–**P**) Coronal sections of the head stained with hematoxylin and eosin. Total 17 animals were examined (11 controls and 6 mutants). The arrow in (**N**) points to the absence of the lower incisor. (**Q**) Summary of the lower incisor phenotype. LI: lower incisor; LM: lower molar; UI: upper incisor; UM: upper molar. Bar: 0.2 mm. Genotypes of the controls shown are *Wnt1-Cre* (**A**,**B**) and *Rspo2^+/−^;Rspo3^fl/+^* (**G**–**K**).

Constitutive knockout of *Rspo3* in mice resulted in mid-gestation lethality from placenta defects [29]. Therefore, we used a floxed allele of *Rspo3* and *Wnt1-Cre* to delete *Rspo3* in neural crest-derived cells [23,24,30]. The *Wnt1-Cre;Rspo3^fl/−^* mutant pups (*Rspo3^NCKO^*) survived after birth at least up to the weaning age. However, they lacked lower incisors, which led to overgrowth of the upper incisors and a difficulty in feeding. Three weeks after birth, we examined four mutants and five littermate controls by Micro-CT (Figure 1A–E). The craniofacial morphology of the mutants was grossly normal. However, in addition to the fully penetrant loss of the lower incisors, the third molars on the lower jaw were missing in some mutants (Figure 1D–F). The same phenotype was reported in mouse mutants with *Lgr4* deletion in the dental epithelium [31]. Thus, it is possible that RSPO3 is the ligand that activates LGR4 for its function in sequential molar development.

We also examined perinatal *Rspo3^NCKO^* mutants (E18.5 or P0) in sections (Figure 1G–P). The lower incisors were affected in all mutants and were either missing or very small (Figure 1K,P,Q). By contrast, the upper incisors and the molars appeared normal at this stage. Because the lower incisor defect was fully penetrant in *Rspo3^NCKO^* mutants, we focused further analyses on this phenotype.

### 3.2. Development of the Lower Incisors Is Arrested at the Bud Stage in Rspo3^NCKO^ Mutants

**Figure 2:**
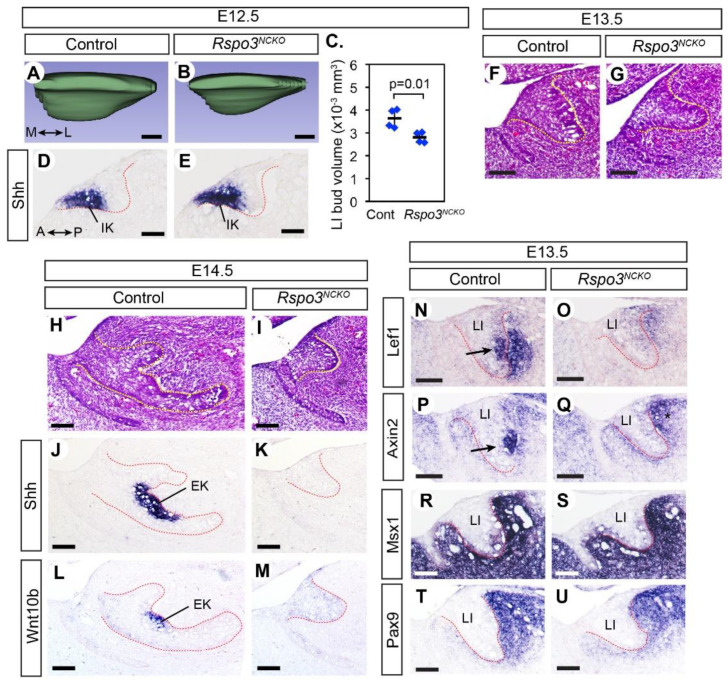
Bud-stage arrest of the lower incisor development in *Rspo3^NCKO^* mutants. (**A**,**B**) 3D rendering of the lower incisor bud at E12.5. The medial (**M**)–lateral (**L**) axis is indicated in (**A**). A total of 8 embryos, one bud per embryo, were examined (4 controls and 4 mutants). (**C**) Comparison of the volume of the lower incisor buds at E12.5. The horizontal bars are the average for each genotype, and the error bars are the standard deviation. Post-hoc power was 0.84. (**D**–**U**) Sagittal sections of the head processed by RNA in situ hybridization (**D**,**E**,**J**–**U**) or stained with hematoxylin and eosin (**F**–**I**). All panels show the lower incisor, and the anterior (**A**)–posterior (**P**) axis is indicated in (**D**). The dotted lines mark the epithelium–mesenchyme boundary. For E14.5, 6 embryos (3 mutants and 3 controls) were examined for each experiment. For E13.5, *Lef1* was examined in 16 embryos (8 controls and 8 mutants) and *Axin2* was examined in 14 embryos (7 controls and 7 mutants). *Pax9* and *Msx1* were each examined in 6 embryos (3 controls and 3 mutants). Arrows in **N** and **P** indicate the expression of *Lef1* and *Axin2* in the nascent enamel knot. Although *Axin2* may appear upregulated in the mesenchyme posterior to the lower incisor bud in the mutant (* in (**Q**)), this is due to a slight difference in the plane of section between (**E**,**G**) and is not a consistent phenotype of the mutant. IK: initiation knot; EK: enamel knot. Bars in (**A**,**B**,**D**,**E**): 0.05 mm; bars in (**F**–**U**): 0.1 mm. Genotypes of the controls shown are *Rspo3^fl/+^* (**A**,**C**,**D**), *Rspo2^+/−^;Rspo3^fl/+^* (**F**,**N**,**P**,**R**,**T**), and *Rspo3^fl/−^* (**H**,**J**,**L**).

At E12.5, the lower incisors of both *Rspo3^NCKO^* mutants and controls reached an early bud stage, but the mutant bud was smaller (Figure 2A–C). During normal development, the incisor makes an early signaling center called an initiation knot, which is marked by the expression of *Shh* and activation of the canonical WNT pathway [32]. The initiation knot appeared unaffected in *Rspo3^NCKO^* mutants (Figure 2D,E and Figure S2). At E13.5–E14.5, the mutant lower incisors were not only small but also missing the enamel knot based on its markers, *Shh* and *Wnt10b* (Figure 2F–M).

Because of the well-documented importance of the canonical WNT signaling in odontogenesis, we examined *Rspo3^NCKO^* mutants for any change in this pathway (Figure 2N–Q, Figures S2 and S3). *Axin2* and *Lef1* are direct transcriptional targets of canonical WNT signaling, and thus both genes are widely used as a read-out of the pathway activity [33,34]. However, we noticed a difference in their expression patterns in the incisors of control embryos (Figure 2N,P). We have made the same observation of a discrepancy in the embryonic head mesenchyme [35], and the reason for this is currently unknown. In the incisor epithelium, both *Lef1* and *Axin2* were strongly expressed at the location of the nascent enamel knot. In the incisor mesenchyme, *Lef1* was strongly expressed next to its expression in the incisor bud, whereas *Axin2* was expressed broadly around the bud at a lower level (Figure 2N,P).

We first compared *Lef1* expression quantitatively in the lower incisors of *Rspo3^NCKO^* mutants and controls at E12.5, but the results were inconclusive (Figure S2). By contrast, at E13.5, most *Rspo3^NCKO^* mutants (7 out of 8) showed a dramatic reduction in *Lef1* expression in the lower incisors, in both the epithelium and the mesenchyme (Figure 2N,O). One mutant retained *Lef1* expression in a lower incisor, which was consistent with the observation that occasionally the lower incisor was present in *Rspo3^NCKO^* mutants at birth (Figure 1Q). This mutant was excluded from other analyses. *Axin2* was greatly downregulated in the lower incisor epithelium of *Rspo3^NCKO^* mutants, but not in the mesenchyme (Figure 2P,Q). Neither *Lef1* nor *Axin2* was significantly affected in the upper incisors or the molars of *Rspo3^NCKO^* mutants (Figure S3). While the reduced expression of *Lef1* and *Axin2* in the mutant lower incisors at E13.5 is consistent with a decrease in canonical WNT signaling, we cannot rule out the possibility that it is an indirect consequence of the failure in enamel knot development because the enamel knot expresses WNT ligands.

We also examined the expression of *Msx1* and *Pax9*, which are expressed in the dental mesenchyme during normal development and are essential to bud-to-cap transition [36,37]. Their expression was unaffected or moderately reduced in *Rspo3^NCKO^* mutants at E13.5 (Figure 2R–U). In addition, even though the lower incisor bud was smaller in the mutants than in controls, the comparison of cell proliferation was inconclusive (Figure S4).

### 3.3. Simultaneous Inactivation of Rspo2 and Rspo3 Leads to Severe Disruption of Craniofacial Development Including Odontogenesis

**Figure 3:**
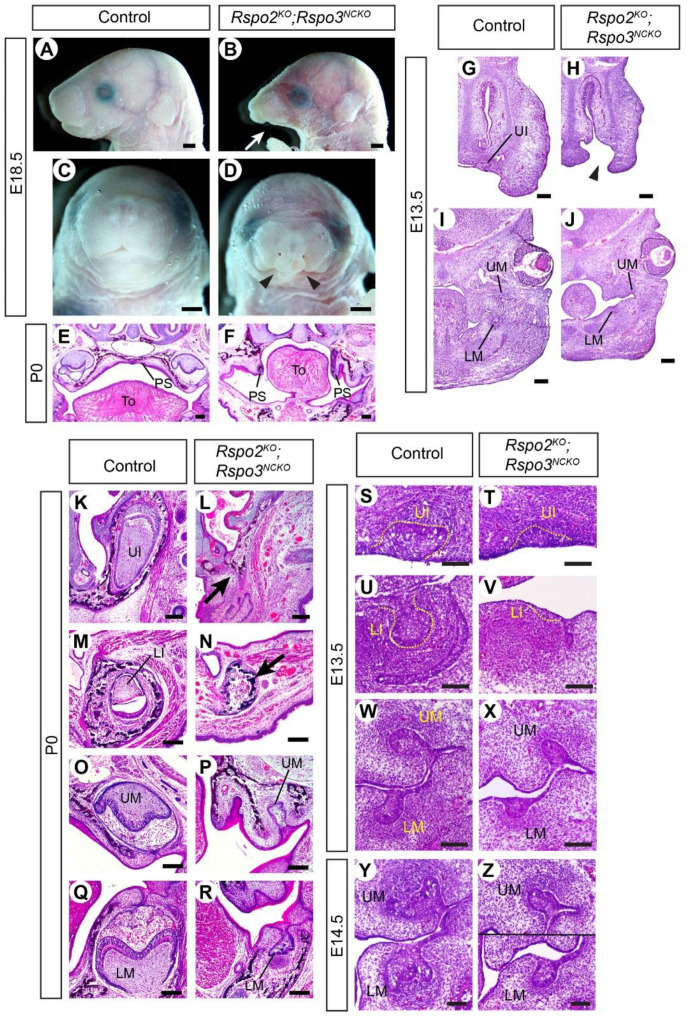
Craniofacial and dental phenotypes of *Rspo2^KO^;Rspo3^NCKO^* mutants. (**A**–**D**) Lateral (**A**,**B**) and frontal (**C**,**D**) views of the head. 15 animals were examined (11 controls and 4 mutants). The arrow in (**B**) points to the hypoplasia of the mandible. Arrowheads in (**D**) point to the cleft lip. (**E**–**Z**) Coronal sections of the head stained with hematoxylin and eosin. A total of 6 animals were examined at each stage (3 controls and 3 mutants). The arrowhead in (**H**) points to the cleft lip. Arrows in (**L**,**N**) indicate the absence of the incisors. PS: palatal shelves, To: tongue. Bars in (**A**–**D**): 1 mm; bars in (**E**–**J**): 0.2 mm; bars in (**K**–**Z**): 0.1 mm. Genotypes of the controls shown are *Rspo2^+/−^;Rspo3^fl/+^* (**A**,**C**,**E**,**M**), *Rspo3^fl/+^* (**G**,**I**), *Rspo3^fl/−^* (**K**,**O**,**Q**), *Wnt1-Cre;Rspo3^fl/+^* (**S**,**U**,**W**), and *Wnt1-Cre;Rspo2^+/−^;Rspo3^fl/+^* (**Y**).

A recent paper has shown that *rspo2* and *rspo3* have overlapping activities in craniofacial development in fish [22]. To uncover the roles of *Rspo2* and *Rspo3* in mice that were potentially masked by an overlap in their functions, we inactivated both genes in the craniofacial mesenchyme in *Wnt1-Cre;Rspo2^−/−^;Rspo3^fl/−^* mutants (*Rspo2^KO^;Rspo3^NCKO^*). All the mutants examined perinatally showed hypoplasia of the mandible and cleft secondary palate. In addition, two mutants had bilateral cleft lip and the other two had unilateral cleft lip (Figure 3A–F). At E13.5, the earliest stage examined, the face of the *Rspo2^KO^;Rspo3^NCKO^* mutants was already grossly abnormal (Figure 3G–H).

Dental phenotype was examined in head sections of three perinatal mutants. The upper incisors and the lower incisors were completely missing, and the upper molars and the lower molars were severely underdeveloped (Figure 3K–R). The mutant molars exhibited some degree of widening and folding of the epithelium in the distal part, which suggested that their development was stalled during the bud-to-cap transition (Figure 3P,R). We examined *Rspo2^KO^;Rspo3^NCKO^* mutants at E13.5 to determine whether they were able to at least initiate incisor development. The upper incisors were present as a bud, although it was smaller than those of control embryos (Figure 3S,T). The lower incisors were barely recognizable as a slight epithelial thickening, which resembled a dental placode or an early bud (Figure 3U,V). Both incisors apparently regressed in *Rspo2^KO^;Rspo3^NCKO^* mutants, so that there were no remnants at E18.5/P0 (Figure 3L,N). In contrast, the mutant molars reached the bud stage at E13.5 and continued to progress in growth and morphogenesis in the following days, albeit with a great delay compared with controls (Figure 3W–Z).

Because the expression of *Rspo2* and *Rspo3* has little overlap during incisor development (Figure S1), the incisor phenotype of *Rspo2^KO^;Rspo3^NCKO^* mutants is likely secondary to defects in early development of the face (Figure 3G–J). Both *Rspo2* and *Rspo3* are expressed in the first pharyngeal arch and the frontonasal process [19,29]. On the other hand, the two *Rspo* genes are co-expressed in the molar mesenchyme at the bud stage [20], and thus the molar phenotype of the double mutant may reflect their direct role in odontogenesis although we cannot rule out the other possibility.

For a comparison, we examined *Rspo2^−/−^;Rspo3^fl/+^* mutants (*Rspo2^KO^*) collected from the same cross as *Rspo2^KO^;Rspo3^NCKO^* mutants at E18.5 and P0. As in previous reports [19,20,21], *Rspo2^KO^* mutants had the diastema teeth in the lower jaw (*n* = 5) and partially penetrant cleft secondary palate (3 out of 5). In addition, we noted a dental phenotype that was not described before (Figure S5). Nonetheless, the defects of *Rspo2^KO^* mutants were much milder than those of *Rspo2^KO^;Rspo3^NCKO^* mutants. Thus, we conclude that the mutations in *Rspo3* and *Rspo2* have synergistic effects on craniofacial and dental development in mice.

Herein, we have shown that without RSPO3, the lower incisors were unable to progress from the bud stage to the cap stage in mouse embryos. This is the first evidence that RSPO signaling promotes the normal process of odontogenesis in mammals. As to why only the lower incisors were affected in *Rspo3^NCKO^* mutants, one of the potential explanations is in the expression pattern of *Rspo1*. At E13.5, there is relatively strong, localized expression of *Rspo1* in the mesenchyme of the upper incisors and both molars, but not of the lower incisors (Figure S1) [20]. We speculate that this difference makes the lower incisors particularly sensitive to the loss of *Rspo3*.

The current study also provides the first description of the phenotypic consequences from the simultaneous loss of *Rspo2* and *Rspo3* during craniofacial development in mammals. A recent publication showed that *Rspo2* and *Rspo3* synergistically regulated craniofacial and dental development in fish [22]. However, they found significant differences between fish and mice in the expression patterns of the two *Rspo* genes, which made it important to examine mouse mutants. While further investigation is necessary to determine the molecular and cellular changes in the mouse double mutants, our data establish that *Rspo3*, along with *Rspo2*, is a crucial regulator of oro-facial development from early stages.

## 4. Conclusions

*Rspo3* is important for development of the lower incisors in mammals, regulating the bud-to-cap transition. Furthermore, *Rspo2* and *Rspo3* have overlapping yet crucial roles in early development of the face in mammals.

## Supplementary Materials

The following are available online at https://www.mdpi.com/article/10.3390/jdb9030031/s1, Figure S1: Expression of *Rspo* genes during normal development of the incisors; Figure S2: Quantitative analysis of LEF1 expression in the lower incisor of *Rspo3^NCKO^* mutants at E12.5; Figure S3: Expression of *Lef1* and *Axin2* in the upper incisor and the molars of *Rspo3^NCKO^* mutants; Figure S4: Cell proliferation analysis in the lower incisor of *Rspo3^NCKO^* mutants; Figure S5: Dental and craniofacial phenotype of *Rspo2^KO^* mutants.
