# Supplementary material for: R-Spondin 3 Regulates Mammalian Dental and Craniofacial Development
Source: J Dev Biol. 2021 Aug 12;9(3):31. doi: 10.3390/jdb9030031 (PMC8395884; doi:10.3390/jdb9030031)
Supplement: Supplementary file 1 [file jdb-09-00031-s001.zip › jdb-1332545-supplementary.pdf]

SUPPLEMENTARY MATERIALS AND METHODS

RNA in situ hybridization

Templates for the anti-sense probes against *Rspo1, Rspo2, Rspo3*, and *Rspo4* were obtained as follows: for *Rspo1* and *Rspo4*, PCR was performed from wild type CD1 mouse tail genomic DNA using primers ACAGTGACCGGTCTCCAGAT (*Rspo1* forward), AATTAACCCTCACTAAAGGGGACGTGTACCACGGATGTGT (*Rspo1* reverse), AAGTGCCTGTAACACACCCTCT (*Rspo4* forward), and AATTAACCCTCACTAAAGGG AAGACAGACTTGCCATTTGGTT (*Rspo4* reverse). The underlined sequence is a binding site for T3 polymerase. For *Rspo2*, a cDNA clone was purchased from Open Biosystems (GenBank accession number BC052844), and PCR was performed from this plasmid using primers GTGTCACCTCATGGCGTTCTCAGC (forward) and AATTAACCCTCACTAAAGGGGGCAAGGAAAGGCTTCGCC (reverse). For *Rspo3*, a cDNA clone was purchased from Open Biosystems (GenBank accession number BC103794), and this plasmid was linearized by EcoRI and transcribed by T7 polymerase.

Immunofluorescence, LEF1 intensity measurement, Ki67 analysis

Immunofluorescence was performed as described before (Jeong and McMahon, 2005). Primary antibodies were rabbit anti-LEF1 antibody from Abcam (ab137872, 1:500) and rat anti-Ki67 antibody from eBioscience (14-5698-80, 1:200). Secondary antibodies were Alexa Fluor568 anti-rat IgG and Alexa Fluor488 anti-rabbit IgG from ThermoFisher Scientific. The nuclei were stained with DAPI.

To quantify LEF1 fluorescence intensity, we opened a gray scale picture of LEF1 staining in FIJI, determined a background level from the tissue itself using a pixel intensity histogram (background appears as a large peak at a low level), and subtracted the background by moving the minimum value in brightness. Subsequently, we demarcated a region of interest as in Figure S2, and measured the total pixel intensity (raw integrated density) and the area. For each embryo, we made these measurements from four sections in which the tooth bud appears the largest, and calculated the average pixel intensity by dividing the sum of raw integrated densities by the sum of the areas.

To determine cell proliferation rates, we demarcated a region of interest as in Figure S4, and manually counted the total cells and Ki67$^+$ cells. For each embryo, we counted the cells from 4 sections (E12.5) or 3 sections (E13.5) in which the tooth bud appears the largest, and calculated the proliferation rate by dividing the sum of Ki67$^+$ cells by the sum of total cells.

Reference

Jeong, J., McMahon, A.P., 2005. Growth and pattern of the mammalian neural tube are governed by partially overlapping feedback activities of the hedgehog antagonists patched 1 and Hhip1. Development 132, 143–154.

Figure S1

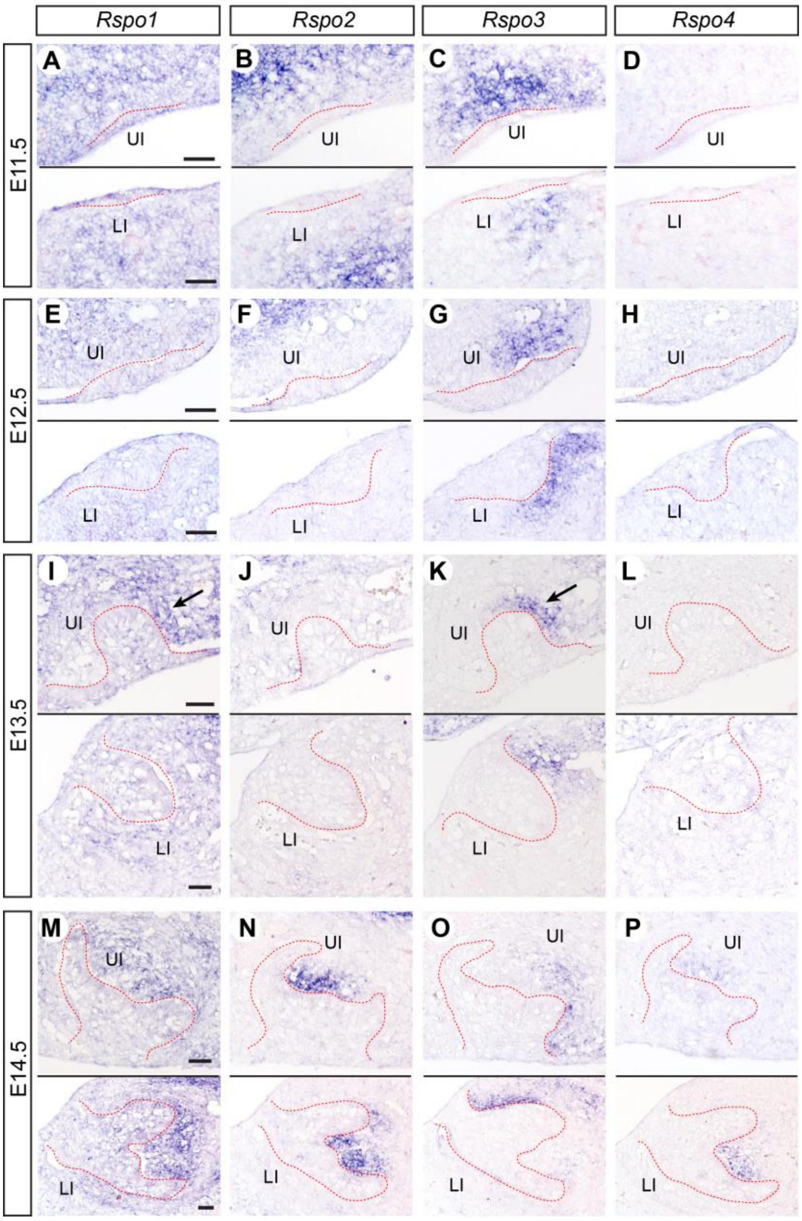

Figure S1. Expression of *Rspo* genes during normal development of the incisors. Sagittal sections through the incisor processed by RNA in situ hybridization. The anterior side is to the left. Red dotted lines mark the boundary between the epithelium and the mesenchyme. *Rspo1* expression was diffuse (A,E,I,M), except for relatively strong expression in the posterior mesenchyme of the upper incisor at E13.5 (arrow in I) and in the dental papilla of both incisors at E14.5 (M). At E11.5, *Rspo2* was faintly expressed in the mesenchyme in the vicinity of the dental lamina (B). *Rspo2* expression was down-regulated at E12.5 and E13.5 (F,J), but appeared again at E14.5 in the dental papilla (N). *Rspo3* was expressed in the dental mesenchyme at all stages examined (C,G,K,O), and it was localized to the posterior mesenchyme at E12.5-E14.5. *Rspo3* was co-expressed with *Rspo1* in the upper incisor mesenchyme at E13.5 (arrow in K). *Rspo4* expression was detected only at E14.5 at a very low level (P). LI: lower incisors, UI: upper incisors. Bar: 0.05 mm. All the samples are wild type C57B6/J. The same embryos were used for the four genes, one embryo per stage, except that *Rspo1* was examined in one more embryo at E13.5.

Figure S2

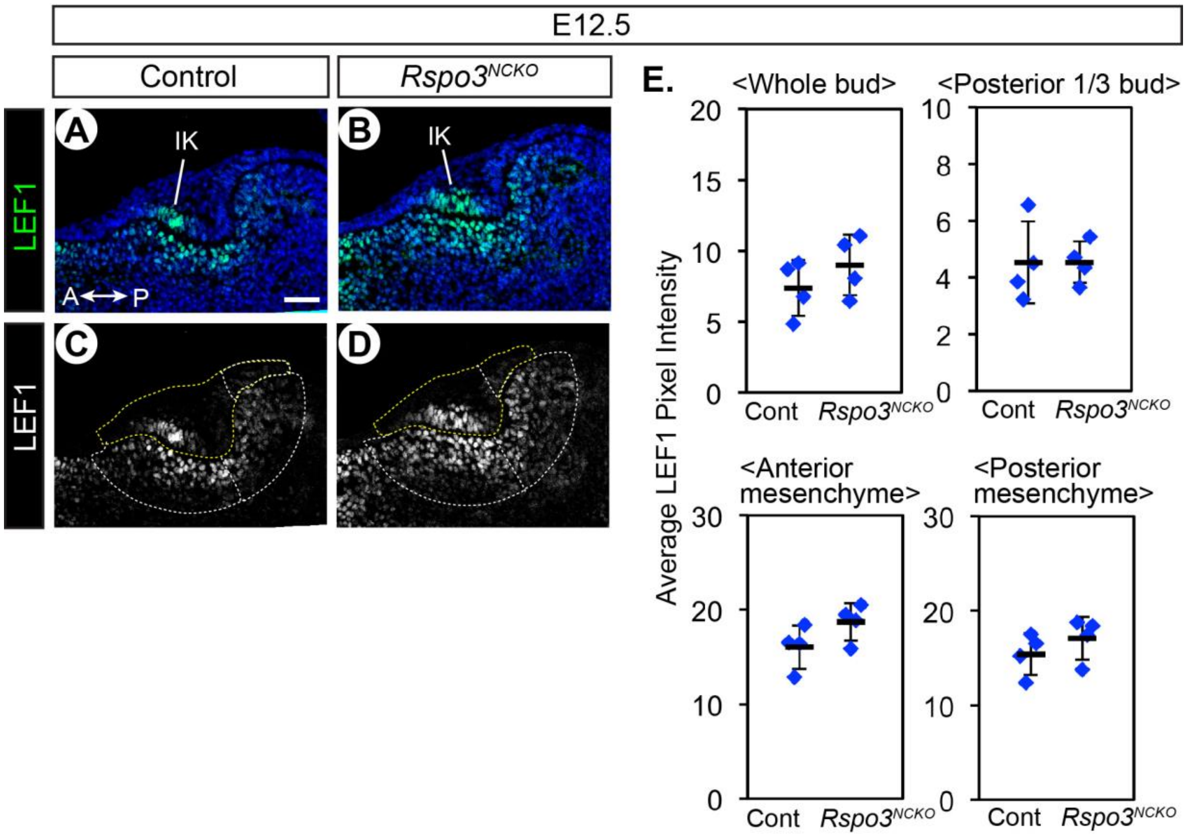

Figure S2. Quantitative analysis of LEF1 expression in the lower incisor of *Rspo3*^{NCKO} mutants at E12.5. (A,B) Sagittal sections through the lower incisor processed by immunofluorescence for LEF1 (green) and stained with DAPI for nuclei (blue). Anterior -posterior axis is indicated in A. (C,D) Green channel images from A and B are marked for the areas where fluorescence intensity was measured for the comparisons in E. The yellow lines demarcate the whole bud. (E) Comparison of LEF1 pixel intensities between controls and *Rspo3*^{NCKO} mutants (n=4/genotype). The horizontal bars are the average for each genotype, and the error bars are standard deviation. The comparisons were inconclusive because post hoc power was <0.8 in all cases. IK: initiation knot. Bar: 0.05 mm. All the controls are *Rspo3*$^{fl/+}$.

Figure S3

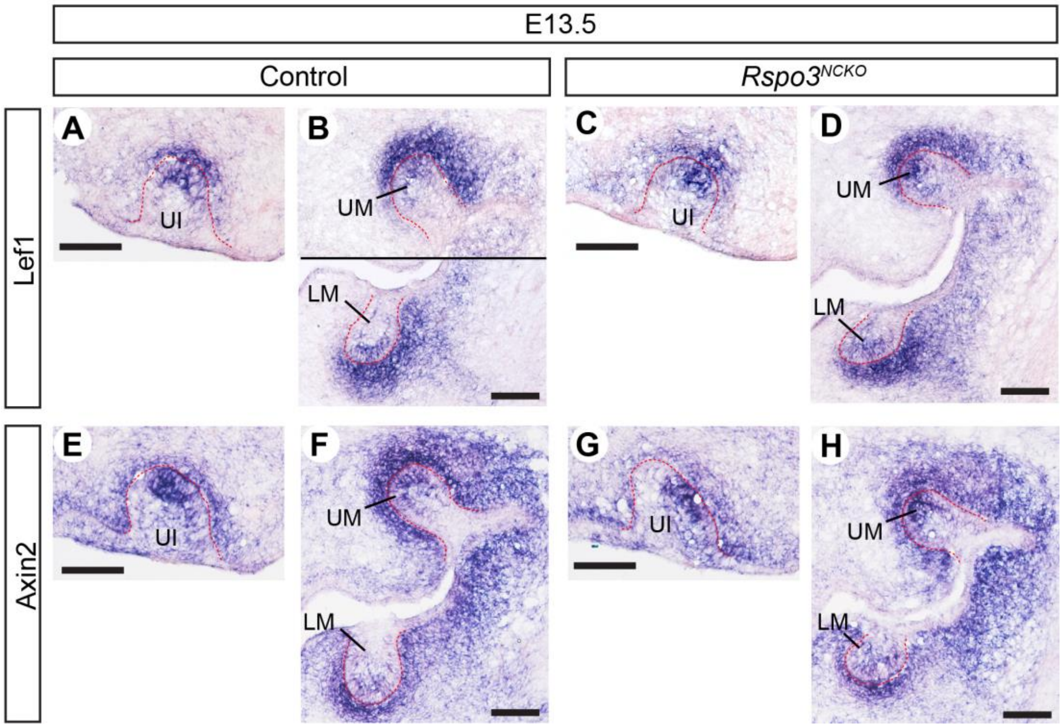

Figure S3. Expression of *Lef1* and *Axin2* in the upper incisor and the molars of *Rspo3*^{NCKO} mutants. Sections processed by RNA in situ hybridization. A,C,E,G are sagittal sections and the rest are coronal. LM: lower molar, UM: upper molar. Bar: 0.1 mm. Genotypes of the controls shown are: *Rspo2*$^{+/-}$;*Rspo3*$^{fl/+}$ (A,E), *Rspo3*$^{fl/-}$ (B), *Rspo3*$^{fl/+}$ (F).

Figure S4

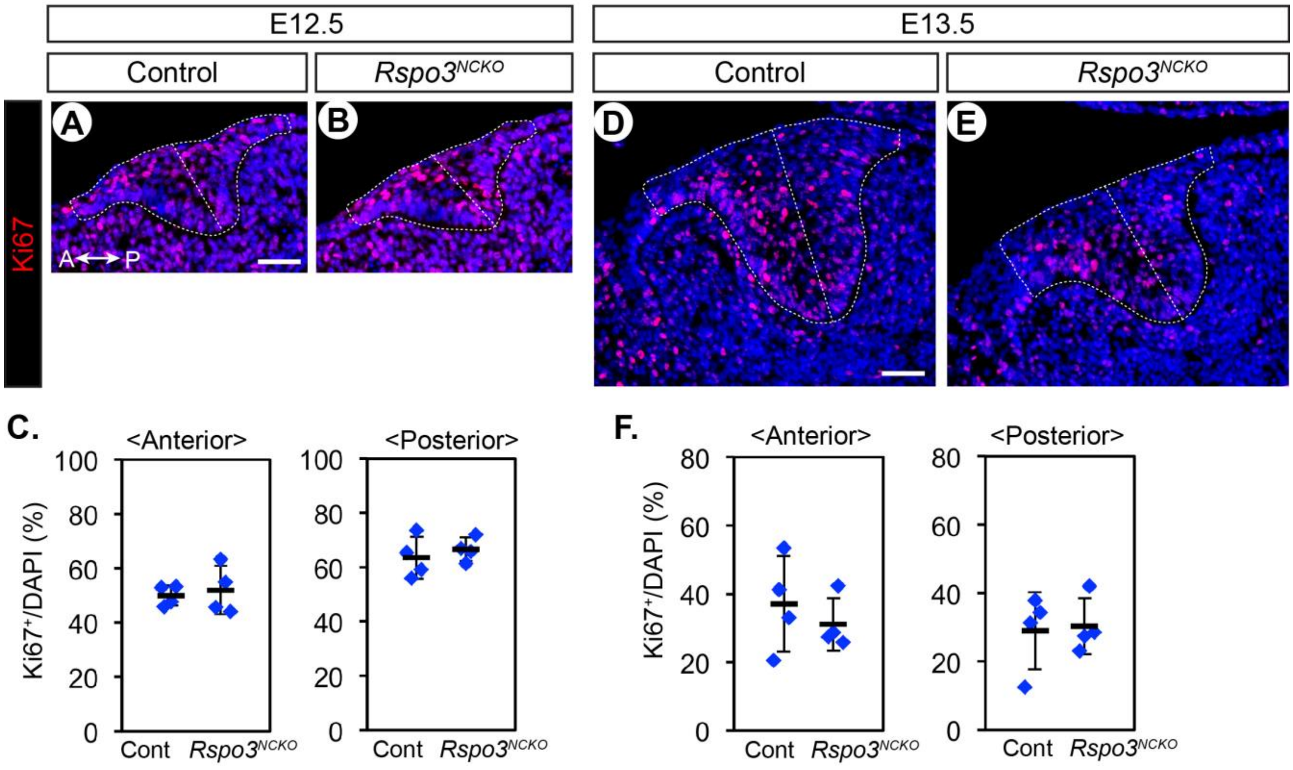

Figure S4. Cell proliferation analysis in the lower incisor of *Rspo3^{NCKO}* mutants. A,B,D,E) Sagittal sections through the lower incisor processed by immunofluorescence for Ki67 (red) and stained with DAPI for nuclei (blue). Anterior-Posterior axis is indicated in A. Dotted lines mark the areas where the cells were counted. C,F) Comparison of cell proliferation rates between controls and *Rspo3^{NCKO}* mutants (n=4/genotype). The horizontal bars are the average for each genotype, and the error bars are standard deviation. The comparisons were inconclusive because post hoc power was <0.8 in all cases. Bar: 0.05 mm. All the controls are *Rspo3$^{fl/+}$*.

Figure S5

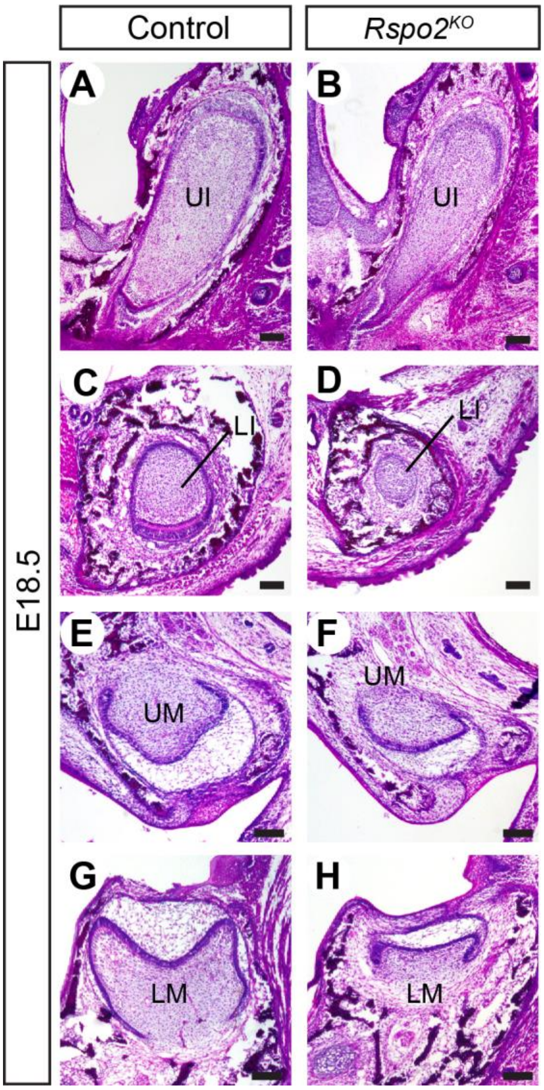

Figure S5. Tooth phenotype of *Rspo2*^{KO} mutants. Coronal sections of the head stained with hematoxylin and eosin. 11 animals (6 controls and 5 mutants) were examined. While the upper incisor appeared normal in *Rspo2*^{KO} mutants (A,B), all the other teeth were significantly under-developed (C-H). A previous report stated that *Rspo2* mutants showed no significant anomalies in the endogenous molars and incisors (Kawasaki et al., 2014), but there are multiple possible explanations for the discrepancy between their results and ours. First, the *Rspo2* mutant allele in Kawasaki et al. was generated by deleting a part of exon2, whereas our mutant allele is additionally missing exon1. Second, the genetic background of the mice is likely different between the two studies, potentially influencing the phenotype. Lastly, our mutants are *Rspo2*$^{-/-}$;*Rspo3*$^{fl/+}$, not *Rspo2*$^{-/-}$;*Rspo3*$^{+/+}$ as in the other study. Although *Rspo3*$^{fl/fl}$ mice are viable and fertile, we do not know for certain whether *Rspo3*^{fl} allele is functionally identical to *Rspo3*$^{+}$ when combined with *Rspo2*$^{-/-}$. Bar: 0.1 mm. All the controls are *Rspo2*$^{+/-}$;*Rspo3*$^{fl/+}$.
